# The glycogen synthase kinase MoGsk1, regulated by Mps1 MAP kinase, is required for fungal development and pathogenicity in *Magnaporthe oryzae*

**DOI:** 10.1038/s41598-017-01006-w

**Published:** 2017-04-19

**Authors:** Tengsheng Zhou, Yasin F. Dagdas, Xiaohan Zhu, Shiqin Zheng, Liqiong Chen, Zachary Cartwright, Nicholas J. Talbot, Zonghua Wang

**Affiliations:** 1 Fujian-Taiwan Joint Center for Ecological Control of Crop Pests, Fujian Agriculture and Forestry University, Fuzhou, 350002 China; 2 Fujian University Key Laboratory for Functional Genomics of Plant Fungal Pathogens, Fujian Agriculture and Forestry University, Fuzhou, 350002 China; 3 School of Biosciences, University of Exeter, Exeter, EX4 4QD UK

**Keywords:** Fungal genetics, Fungal pathogenesis

## Abstract

*Magnaporthe oryzae*, the causal agent of blast disease, is one of the most destructive plant pathogens, causing significant yield losses on staple crops such as rice and wheat. The fungus infects plants with a specialized cell called an appressorium, whose development is tightly regulated by MAPK signaling pathways following the activation of upstream sensors in response to environmental stimuli. Here, we show the expression of the Glycogen synthase kinase 3 (GSK3) *MoGSK1* in *M*. *oryzae* is regulated by Mps1 MAP kinase, particularly under the stressed conditions. Thus, *MoGSK1* is functionally characterized in this study. MoGsk1 is functionally homologues to the *Saccharomyces cerevisiae* GSK3 homolog MCK1. Gene replacement of *MoGSK1* caused significant delay in mycelial growth, complete loss of conidiation and inability to penetrate the host surface by mycelia-formed appressorium-like structures, consequently resulting in loss of pathogenicity. However, the developmental and pathogenic defects of *Δmogsk1* are recovered *via* the heterologous expression of *Fusarium graminearum* GSK3 homolog gene *FGK3*, whose coding products also shows the similar cytoplasmic localization as MoGsk1 does in *M*. *oryzae*. By contrast, overexpression of *MoGSK1* produced deformed appressoria in *M*. *oryzae*. In summary, our results suggest that MoGsk1, as a highly conservative signal modulator, dictates growth, conidiation and pathogenicity of *M*. *oryzae*.

## Introduction

Phytopathogenic fungi pose a constant threat to the global food security^1^. *Magnaporthe oryzae* (*Pyricularia oryzae*), the causal agent of rice blast disease, is the most devastating plant pathogen on rice and causes up to 30% yield loss of rice every year^2^. Beside rice, it also causes yield losses in other cereals such as wheat, barley and finger millet^3–5^. The infection cycle of the fungus starts when the three-celled conidia land on the leaf surface. Upon landing on cuticle, the spores form a polarized germ tube. Recognition of environmental cues such as surface hydrophobicity and toughness induces swelling at the tip of germ tube, which then differentiates into the specialized infection cell called an appressorium. The appressorium generates enormous turgor pressure by accumulating glycerol with the help of melanised cell wall. This pressure is converted into mechanical force to penetrate the plant surface, followed by further invasive hyphal expansion to colonize plant tissues^6^.

Perception of cues on leaf surface is the key to initiation of cellular responses mediating appressorium development. Coordination of these morphogenetic events is governed by conserved MAP kinase pathways^7^. In *M*. *oryzae*, there are three MAPK pathways namely Pmk1, Hog1 and Mps1. Pmk1 is a central regulator of appressorium development and the *pmk1* mutant is unable to form appressoria and cause disease^8^. Hog1 is dispensable for fungal virulence but is necessary for adaptation to osmotic stress^9^. In contrast to the essential role of Pmk1 in appressorium formation, Mps1 is required for appressorium penetration of plant surface via modulating the fungal cell wall integrity^10^. Beside its role in pathogenesis, Mps1 is active in conidiation and cellular stress responses in *M*. *oryzae* ^10^. Identification of downstream effectors of the Mps1 pathway is critical for understanding the signaling network required for fungal pathogenesis. To date two effectors of the Mps1 pathway have been characterized in *M*. *oryzae*. Mig1 is a MADS-box transcription factor, which has been shown to interact directly with Mps1 in the yeast two-hybrid assay. The *mig1* mutants in *M*. *oryzae* are able to form appressoria that penetrate the plant surface and elaborate into primary infectious hyphae but fail to expand to the neighboring cells, causing loss of pathogenicity^11^. The *M*. *oryzae* Swi6 is an APSES family transcription factor that also physically interacts with Mps1 in both *in vivo* and *in vitro* assays. The *Δswi6* mutant forms deformed and non-melanized appressoria that are unable to penetrate plant surface due to the impaired cell wall integrity^12^.

In addition to targeting transcriptional factors in the nucleus, the Mps1 homolog in *Saccharomyces cerevisiae* Mpk1 has been shown to function in the cytoplasm mediating a signal cascade in response to environmental stress^13^. Glycogen synthase kinase 3 (GSK3) is a unique kinase that contains well-conserved protein structures and phosphorylation properties but with varied roles in mediating a wide range of signal pathways in eukaryotic organisms^14^. GSK3 was initially identified as a kinase that phosphorylates glycogen synthase and causes its inhibition in rabbit cells^15^. A growing body of literatures have stated that GSK3 is able to phosphorylate various targeting proteins in regulation of several cellular events and subjected to phosphorylation by other kinases in response to different signals^16^. One example is the phosphorylation of GSK3 at the N-terminal serine residue by the MAPK cascade and caused its inhibition in response to insulin-like growth factors^17^, subsequently leading to the de-phosphorylation and activation of the GSK3 downstream targeting proteins. In facts, as a ubiquitous kinase, the inhibition of GSK3 that unleashes the activities of the downstream targeting proteins, underpins its regulatory function^16^. One of the main roles for GSK3 is in regulation of cellular response to the extracellular stimuli. In *S*. *cerevisiae*, four homologs to the mammalian GSK3 were identified including the MCK1 that is responsible for the activation of the stress-responsive transcriptional activator MSN2 under various environmental stresses^18^. MCK1 is also a key regulator of the cell cycle progression promoting the timing degradation of the CDC6p as essential component of the pre-replicative complex (pre-RC)^19^. In the plant pathogen *Fusarium graminearum*, one GSK3 homolog Fgk3 was identified and proven to be required for proper growth, conidiogenesis, sexual production and pathogenesis^20^. The multiple phenotypic effects of the *Δfgk3* mutant suggest that Fgk3 is a conservative regulator involved in various aspects of fungal development. The study also showed Fgk3 physically interacts with MSN2 and controls the expression of stress response-related genes under cold, heat, and salt stresses.

In an initial screen for possible downstream cytoplasmic targets of the Mps1 MAP kinase pathway in *M*. *oryzae*, we found the hydrophobin gene *MPG1* and an uncharacterized glycogen synthase kinase 3 (GSK3) homolog *MoGSK1* were differentially expressed in the absence of *MPS1* and in response to environmental stresses. Here, we studied the functions of the GSK3 homolog in *M*. *oryzae* (MoGsk1) transcriptionally regulated by the MPS1 MAPK pathway and revealed its critical role in fungal development and plant infection. Deletion of *MoGSK1* causes remarkable reduction of mycelial growth and loss of asexual spore production necessary for normal spread and infection. Moreover, inoculation using the mycelial plugs of *Δmogsk1* showed a failure of appressorium-like structure penetration and the mutant is unable to infect barley or rice host plants. The complementation by the *Fusarium gramienearum* homolog *FGK3* fully restores the growth and pathogenicity of *Δmogsk1*, and the gene product shows the same cytosolic localization as MoGsk1. This indicates that the two GSK3 homologs in *M*. *oryzae* and *F*. *graminearum* shares highly conserved functions. Overexpression of *MoGSK1* caused deformed appressoriain, possibly due to misregulation of morphogenesis checkpoint. Taken together, our results proves that MoGsk1 is a central signal regulator involved in the stress-responsive mechanism and controls multiple aspects of development in *M*. *oryzae*, from growth, asexual production to pathogenesis.

## Results

### Identification of MPG1 and MoGsk1 as targets of the Mps1 MAP kinase pathway

**Figure 1:**
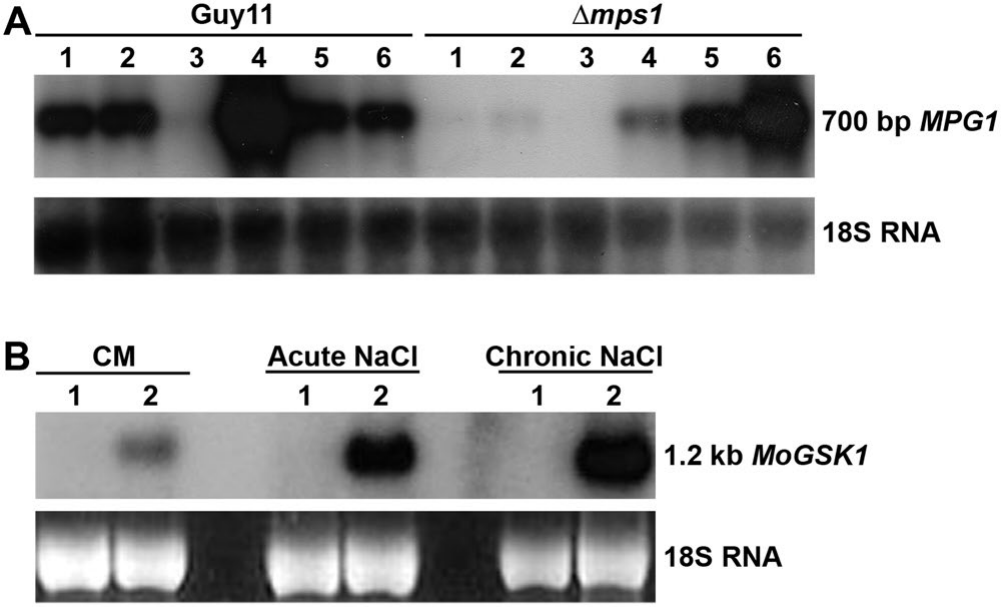
Differential expression of *MPG1* and *GSK1* in the *Δmps1* mutant compared to Guy11. (**A**) RNA gel blot analysis of *MPG1* in Guy11 and *Δmps1* in response to different growth conditions: CM medium (Lane 1), CM + 0.4 M NaCl (acute treatment) (Lane 2), CM + 0.4 M NaCl (chronic treatment) (Lane 3), MM medium − nitrate salts (Lane 4), MM − glucose (Lane 5), and sterile distilled water (Lane 6). (**B**) RNA gel blot analysis of *MoGSK1* in Guy11 (Lane 1) and *Δmps1* (Lane 2) in growth conditions of CM, CM + 0.4 M NaCl (acute treatment) and CM + 0.4 M NaCl (chronic treatment). Total RNA was extracted from mycelia cultured in CM for 48 hr then transferred to different liquid media indicated above for another 24 hr growth. For chronic NaCl treatment, mycelia were obtained after growth in CM + 0.4 M NaCl for 48 hr then transferred to fresh 0.4 M NaCl medium for another 24 hr growth.

In an effort to identify downstream regulated targets of the Mps1 MAP kinase pathway based on expressional differentiation between the *MPS1* (MGG_04943.6) mutant and the wild type *M*. *oryzae* Guy11, *MPG1* (MGG_10315.6) and the sole GSK3 homolog in *M*. *oryzae MoGSK1* (MGG_12122) showed differentiated expression patterns under various stress conditions based on the Northern blot analysis. In the *Δmps1* mutant, expression of *MPG1* was down-regulated compared to Guy11 under conditions of complete medium (CM), acute and chronic salt stresses, and minimal medium (MM) without nitrate salts (Fig. 1A) (lane 1–4). Expression of *MPG1* was however up-regulated during hypo-osmotic stress (water) (Fig. 1A) (lane 6). No difference in the *MPG1* expression was observed during carbon starvation in MM medium without carbon source (Fig. 1A) (lane 5). The expression of *MoGSK1* was up-regulated in the *Δmps1* mutant in CM medium compared to Guy11. The transcriptional induction of *MoGSK1* appeared to be much more prominent when mycelia were exposed to acute and chronic salt stress in the *Δmps1* mutant compared to Guy11 (Fig. 1B). Interestingly, the *MoGSK1* transcript was hardly detectable in Guy11 under all *in vitro* conditions tested (Fig. 1B), suggesting a suppressing effect of Mps1 on the *MoGSK1* transcription in the mycelial stage of the wild type strain. The importance of hydrophobin Mpg1 on infection-related development in *M*. *oryzae* has been well described^21, 22^, here we decided to focus on functional characterization of *MoGSK1*.

**Figure 2:**
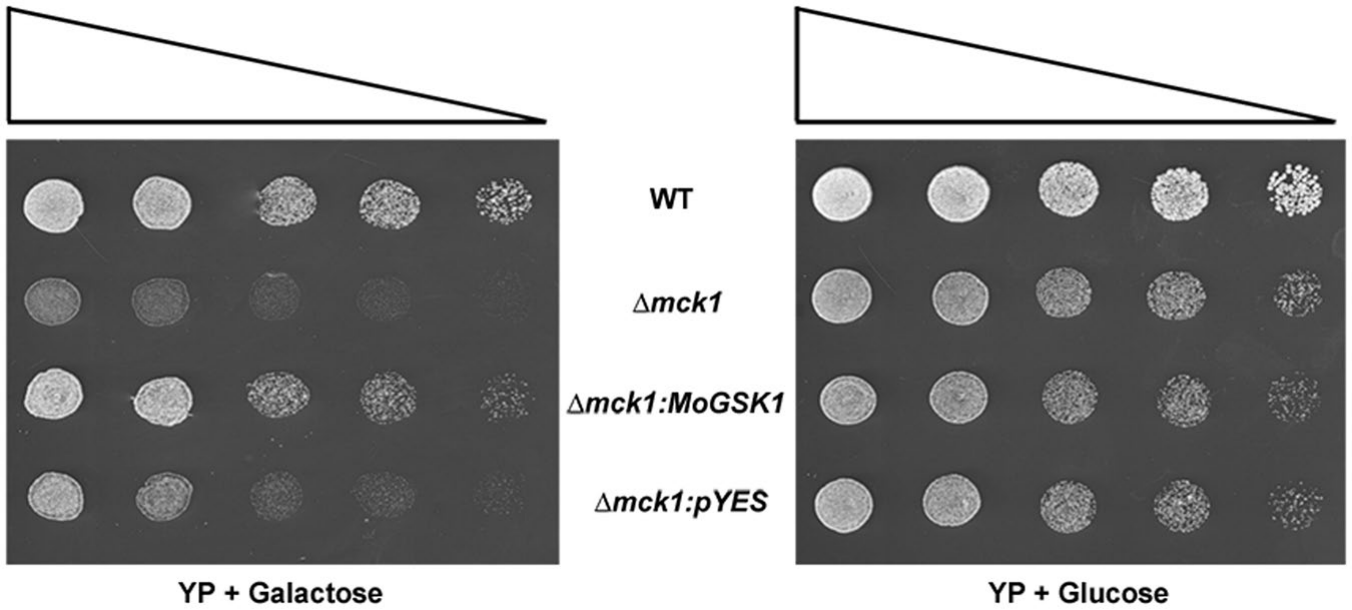
*M*. *oryzae MoGSK1* can functionally complement the *Δmck1* mutant of *S*. *cerevisiae*. Yeast strains were grown at 12 °C for 14 days on galactose or glucose-supplemented YP medium. Plates were inoculated with 10 μl droplets containing 1 × 10^6^, 5 × 10^5^, 1 × 10^5^, 5 × 10^4^, or 1 × 10^4^ cells/ml and left to grow for 14 days before examination. Triangles indicate the decreasing concentration of yeast cells. BY1471 (WT) is the wild type *S*. *cerevisiae* strain. *Δmck1* is the yeast mutant sensitive to cold condition. The *Δmck1:MoGSK1* transformant is expressing the *M*. *oryzae MoGSK1* under the *S*. *cerevisiae* galactose-inducible *GAL1* promoter. The negative control is the *Δmck1* mutant transformed with the empty *pYES* vector.

To determine the phylogenetic relationship of MoGsk1 with other fungal members of GSK3, protein alignments were performed using both identified and predicted fungal GSK3 members from *S*. *cerevisiae*, *F*. *graminearum*, *Colletotrichum graminicola* and *Ustilago maydis*. MoGsk1 is 41% identical to the *S*. *cerevisiae* Mck1, 91% identical to the *F*. *graminearum* Fgk3, 92% identical to the predicted *C*. *graminicola* CgGsk1 and 69% identical to the predicted *Ustilago maydis* UmGsk1 (Fig. S1). Previous studies showed disruption of the *S*. *cerevisiae MCK1* causes increased cold sensitivity^23^. To test whether MoGsk1 is a functional GSK3 homolog, we expressed the *MoGSK1* under the *S*. *cerevisiae* galactose-inducible *GAL1* promoter in the yeast *Δmck1* mutant. Under the induction of galactose as the carbon source, cold tolerance of the *Δmck1* mutant was recovered by MoGsk1 when grown at 12 °C for 14 days (Fig. 2).

### MoGsk1 is essential for normal mycelial growth and conidiation

**Figure 3:**
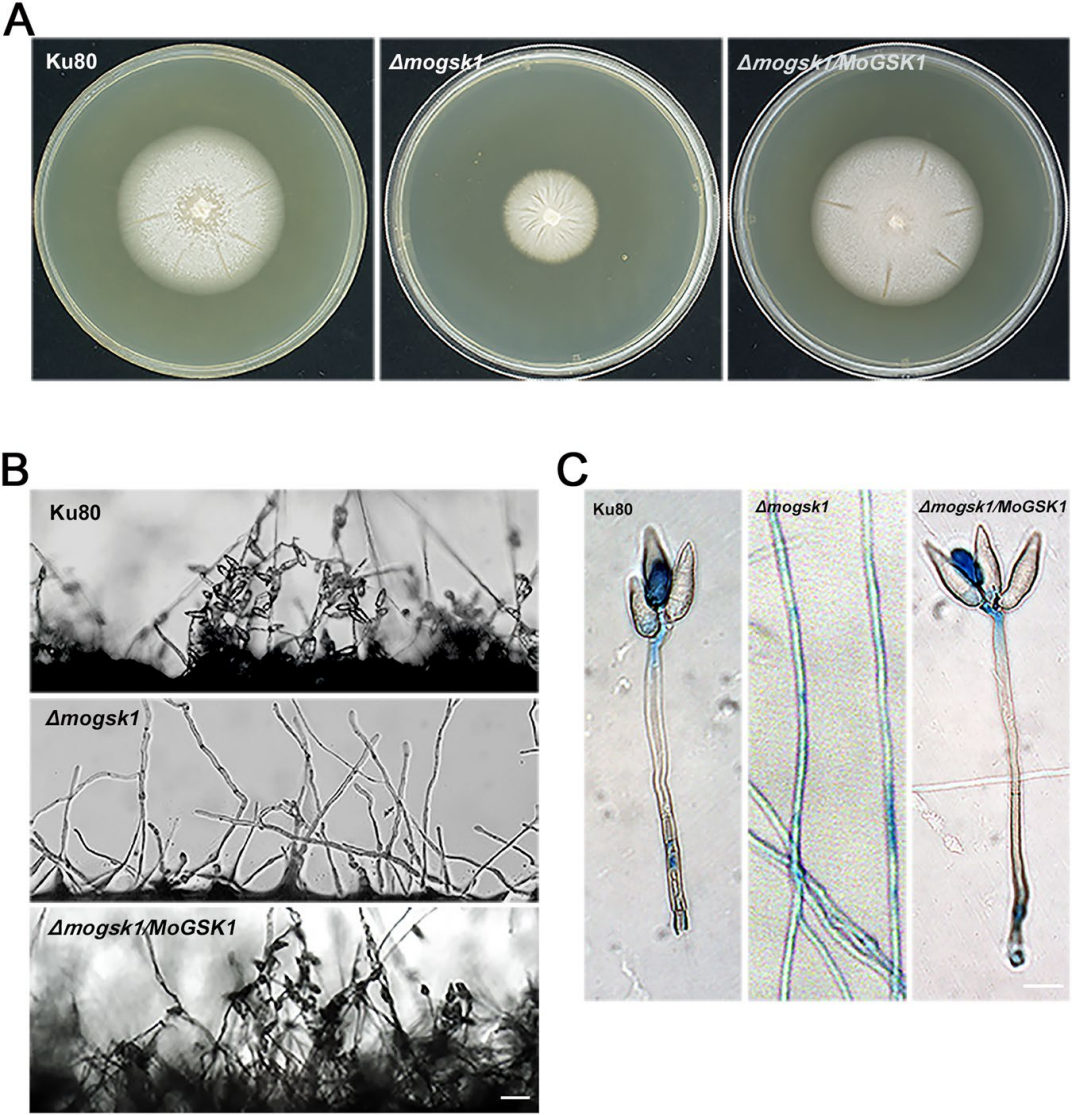
Colony morphology and conidia formation of the *Δmogsk1* mutant. (**A**) Colonies of Ku80, *Δmogsk1* and *Δmogsk3/MoGSK1* grown on complete medium (CM) at 26 °C. Photos were taken at 7 days after inoculation. (**B**) Comparison of conidia formation under light microscope between Ku80, *Δmogsk1* and *Δmogsk1/MoGSK1* after 48 hours induction at 26 °C on glass slides Bar = 50 μm. (**C**) Microscopic observation of aerial structures stained by lactophenol aniline blue. Aerial hyphae were stained blue, while conidiophore stalk remained grey. Bar = 10 μm.

To functionally characterize MoGsk1, we performed targeted gene replacement of *MoGSK1* in the wild type *M*. *oryzae* Ku80 strain (Fig. S2). The *Δmogsk1* mutant displayed remarkable reduction of growth on CM medium (Fig. 3A). Indeed, *Δmogsk1* showed a remarkable reduction of growth rate compared to Ku80. No conidia were detected in *Δmogsk1* when cultured on oat media under constant light exposure (Fig. 3B). The lactophenol aniline blue was used to detect the fungal conidiophore that resists the staining. The staining of fungal aerial structures showed only aerial hyphae were detected and lack of conidiophore in *Δmogsk1*, while the conidiophore stalks in Ku80 remained grey (Fig. 3C). However, defects in mycelial growth and conidiation were fully recovered in the complementation strain *Δmogsk1/MoGSK1* when the *MoGSK1* gene was expressed in *Δmogsk1* under the native promoter. There results suggest that, similar to the GSK3 homologs in *S*. *cerevisiae* and *F*. *graminearum*, MoGsk1 plays a critical role in fungal growth and conidiation^20, 24^.

### MoGsk1 is required for hypha-driven appressorium mediated pathogenesis

**Figure 4:**
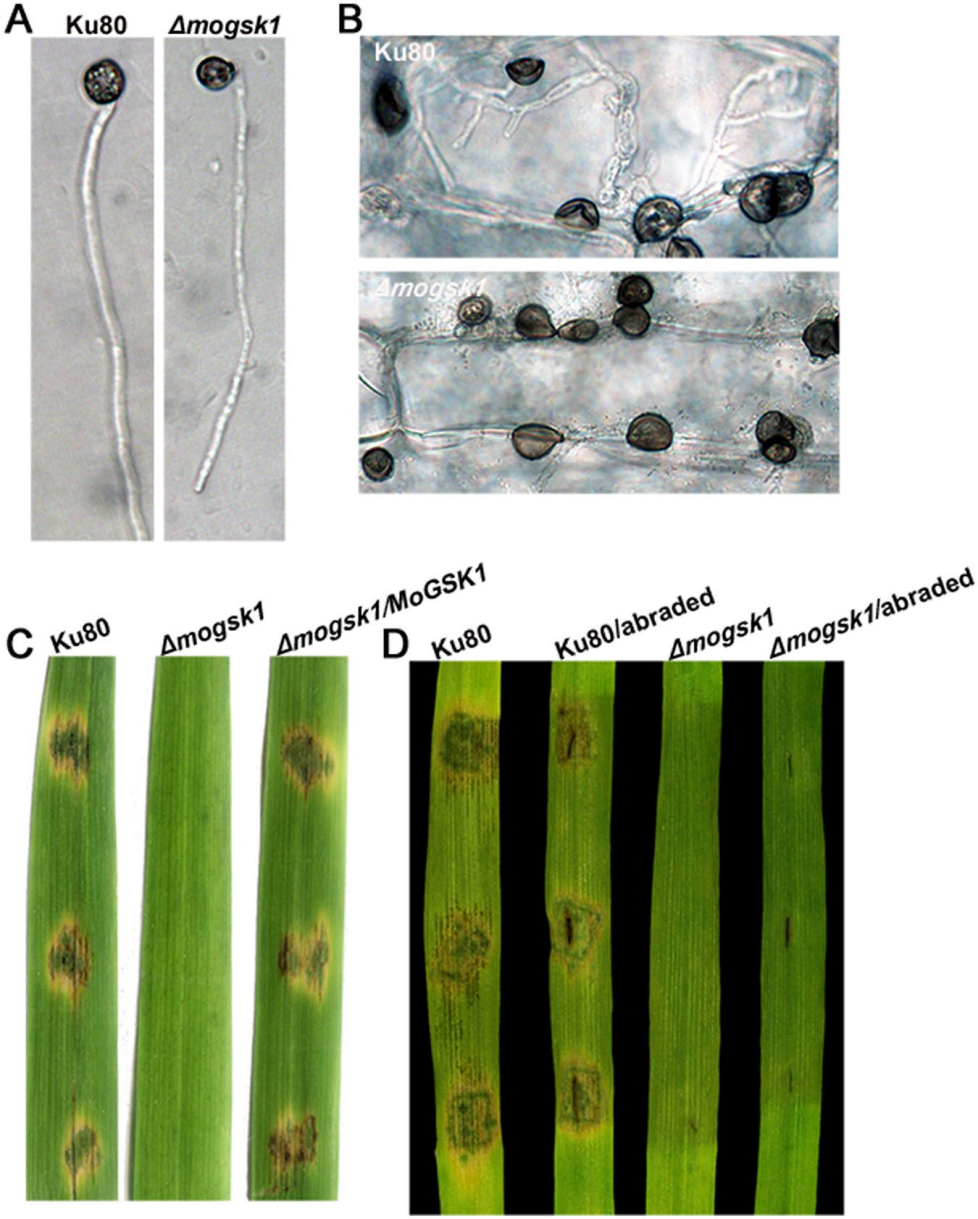
Plant infection assays and microscopic observation on infection process of the *Δmogsk1* mutant. (**A**) Appressoria of Ku80 and *Δmogsk1* were induced at the hyphal tips following 48 h inoculation on hydrophobic cover slips at a moisture chamber at room temperature. Bar = 10 μm. (**B**) Microscopic observation on mycelial plug inoculated area on unwounded barley leaf tissues at 48 hr post inoculation. Bar = 10 μm. (**C**) Equal amounts of mycelial plugs from Ku80, *Δmogsk1* and *Δmogsk1/MoGSK1* were inoculated on 15-day-old rice seedlings (CO39). Photos were taken post 5-day inoculation. (**D**) Disease symptoms on wounded and unwounded 7-day-old susceptible barley seedlings induced by mycelia plugs of Ku80 and *Δmogsk1* were photographed post 5-day inoculation.

**Figure 5:**
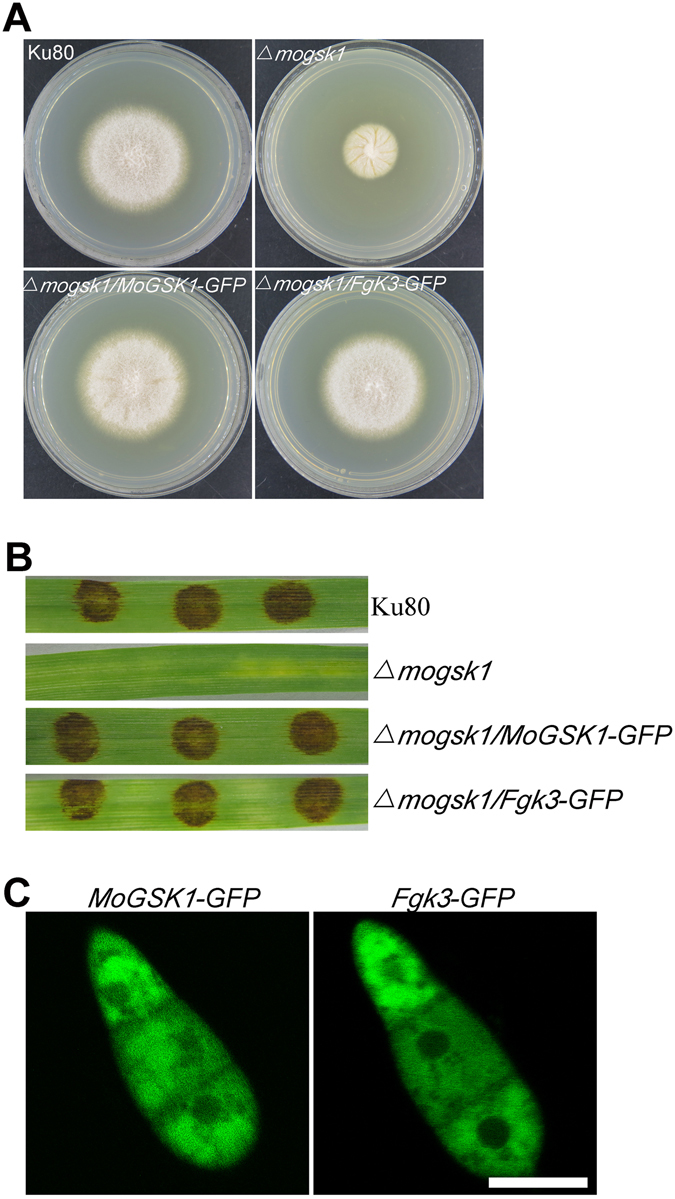
Subcellular localization and complementation of MoGsk1 and Fgk3 in *M*. *oryzae*. (**A**) Colonies of Ku80, *Δmogsk1*, *Δmogsk1/MoGSK1*-*GFP* and *Δmogsk1/Fgk3*-*GFP* grown on CM medium at 26 °C. Photos were taken at 7 days after inoculation. (**B**) Equal amounts of mycelial plugs from Ku80, *Δmogsk1*, *Δmogsk1/MoGSK1*-*GFP* and *Δmogsk1/Fgk3*-*GFP* were inoculated on 7-day-old barley seedlings to observe pathogenic development. Photos were taken post 5 days inoculation. (**C**) Conidia from transformants *Δmogsk1/MoGSK1-GFP* and *Δmogsk1/Fgk3*-*GFP* were examined by epifluorescence (GFP) microscopy. Bar = 10 μm.

It has been revealed that mycelial tips can form appressorium-like structures able to penetrate plant surface in *M*. *oryzae* ^25^. To test the role of MoGsk1 for hypha-driven appressorium development, three day-old mycelial plugs from CM medium were sliced and incubated on hydrophobic cover slips to induce appressorium-like structures. After 48 hr induction, comparable domed and melanized appressorium-like structures were induced at mycelial tips in Ku80 and *Δmogsk1* (Fig. 4A). The appressorium-like structures were also observed by inoculating mycelial plugs on barley leaves in both Ku80 and *Δmogsk1* for 48 hr (Fig. 4B). However, appressorium-like structures developed by *Δmogsk1* failed to penetrate the barley leaf surface while extensive primary invasive hyphae were detected on Ku80-inoculated barley leaf surface (Fig. 4B). The pathogenicity assay was conducted on detached rice and barley leaves using mycelial plug inoculation. After 5-day inoculation, blast disease lesions were observed on Ku80-inoculated leaf surface but absent on *Δmogsk1*-inoculated leaf surface (Fig. 5C and D). Even after abrasion of barley leaf surface, *Δmogsk1* was still not virulent (Fig. 5D). Importantly, pathogenicity was completely recovered in the complementation strain *Δmogsk1/MoGSK1* inoculated on rice seedlings under the same conditions (Fig. 4C).

Lithium has been shown as a direct inhibitor of GSK3 activity in mammalian cells^26^. Since no conidia were detected in the *Δmogsk1* mutant, application of LiCl with increase concentrations (3–10 mM) was performed on wild type Guy11 conidia during the germination on hydrophobic cover slips for 16 hr to test the role of GSK3 activity on appressorium development. A gradual inhibition of appressorium formation was found with a total inhibition at 10 mM without affecting the conidial germination (Fig. S3), indicating an important role of MoGsk1 activity in appressorium development.

### Subcellular localization of MoGsk1 and functional conservation with Fgk3

To examine the subcellular localization, an expression construct of *MoGSK1* directed by the native promoter and fused with GFP at the C-terminus was created and transformed into the *Δmogsk1* mutant. The phenotypic analysis showed that mycelial growth, conidiogenesis, pathogenicity in the transformant *Δmogsk1/MoGSK1-GFP* was rescued (Fig. 5A and B), suggesting the expression construct is functional. Epifluorescence microscopic examination of conidia showed cytoplasmic presence of MoGsk1-GFP but absence in the nuclei (Fig. 5C), which was consistent with the finding of Fgk3 mainly localizing to the cytoplasm at the conidial stage^20^. To reveal the functional relatedness between MoGsk1 and Fgk3, we expressed *FGK3* under the *MoGSK1* native promoter fused with GFP at C-terminus in *Δmogsk1*. As expected, *FGK3* complemented *MoGSK1*, and the growth, conidogenesis and pathogenic deficiencies in *Δmogsk1* were recovered as observed in *Δmogsk1/MoGSK1-eGFP* (Fig. 5A and B). The subcellular observation showed that Fgk3 localized in conidial cytoplasm (Fig. 5C). These results indicated that the GSK3 homologs in phytopathogenic fungi were functionally conserved.

### The effect of the *MoGSK1* overexpression on appressorium morphology

**Figure 6:**
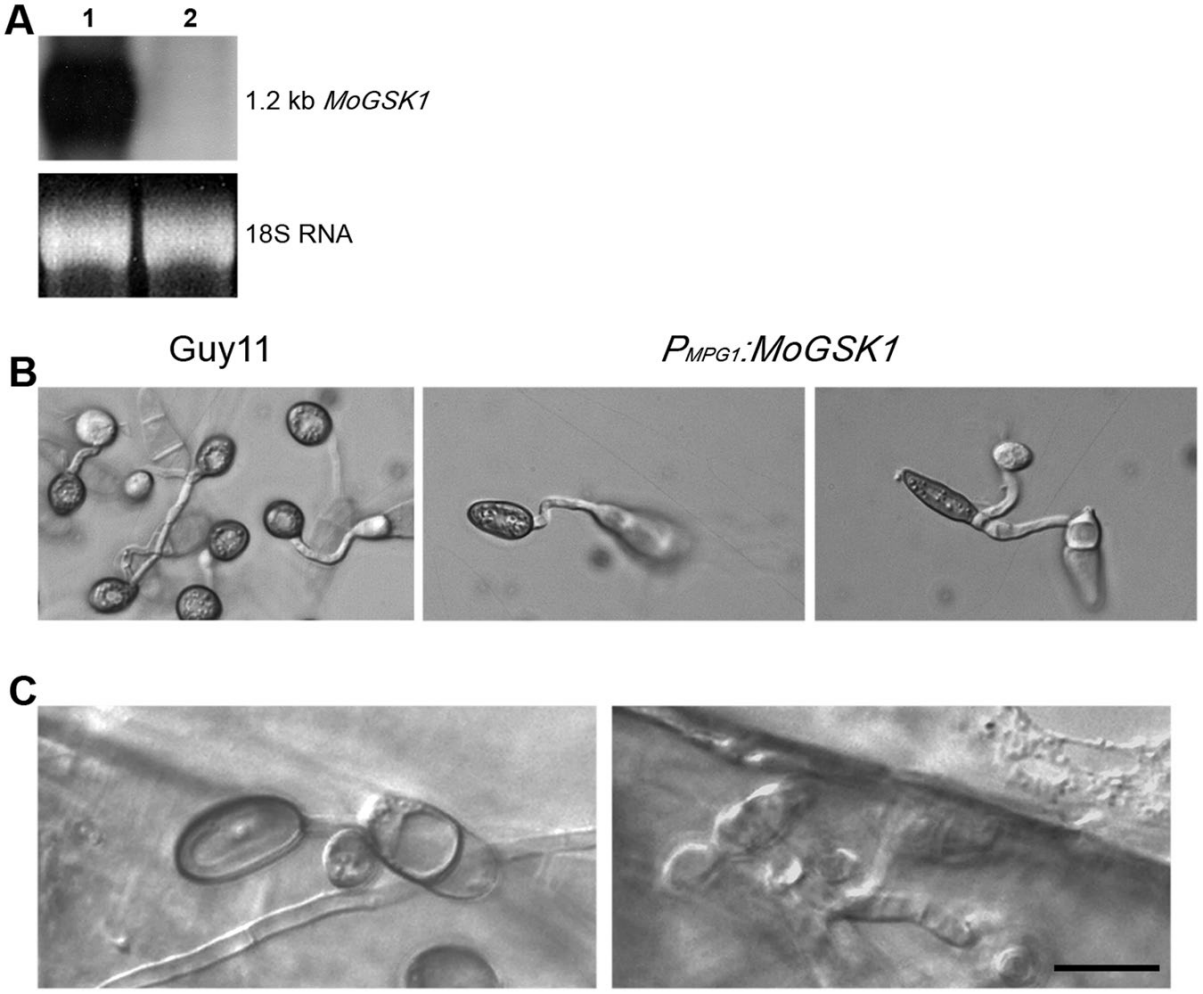
Over-expression of *MoGSK1* affects appressorium morphogenesis in *M*. *oryzae*. (**A**) RNA gel blot showing induction of *MoGSK1* (Line 1) in the transformant expressing *P*_*MPG1*_:*MoGSK1* compared to Guy11. (**B**) Microscopic observation of appressorium morphology induced on hydrophobic cover slips for 24 hr in the transformant expressing *P*_*MPG1*_*:MoGSK1*. Bar = 10 μm. (**C**) Penetration assay to demonstrate pathogenicity of the *MoGSK1* overexpression strain. Appressorium formation (24 hr) and penetration hyphae (48 hr) developed on plant surface are shown in left and right hand panels. Bar = 10 μm.

The regulatory function of MoGsk1 is strictly associated with its spatial and temporal pattern^27^. Here, we aimed to explore the effect of the overexpressing *MoGSK1* in appressorium development and pathogenicity. *MPG1* has been shown to be highly induced during appressorium maturation^28^, therefore *MoGSK1* was expressed under the *MPG1* promoter in the Guy11 strain. The expression level of *MoGSK1* in transformant *P*_*MPG1*_*:MoGSK1* was highly upregulated compared to Guy11 (Fig. 6A). Interestingly, the *MoGSK1* overexpression resulted in production of elongated appressoria on hydrophobic cover slips (Fig. 6B). However, these appressoria were fully capable of penetrating the plant surface and differentiate into invasive hyphae that displayed similar aggression as the Guy11 strain (Fig. 5C).

## Discussion

The SLT2 MAP kinase-dependent cell wall integrity signaling pathway in *S*. *cerevisiae* is involved in coordination of environmental stresses on the cell surface to cellular responses and development^29^. Upon activation, SLT2-mediated signaling can target at transcriptional factors like Rlm1 and Swi6p that further control the expression of genes in charge of cell wall compound synthesis^30, 31^. In *M*. *oryzae*, Mps1 as the functional homolog of SLT2 controls cell wall integrity^10^. The *Δmps1* mutant develops cell walls sensitive to degradation enzymes, produces less conidia and forms appressoria incapable of penetrating the plant surface. Both Rlm1 and Swi6 are identified as nuclear targets of Mps1 signaling pathway in regulation of cell wall integrity, cellular response and pathogenicity in *M*. *oryzae* ^11, 32^. Here, we showed two genes (*MoGSK1* and *MPG1*) with cytoplasmic location of their encoding products were targeted by the Mps1 signaling pathway in the Northern blot-based differential expression-screening assay (Fig. 1). Unexpectedly, higher expression of *MoGSK1* was detected in the *Δmps1* mutant particularly responding to hyper-osmotic stress, indicating an unusual role of *MoGSK1* in the signaling transduction. In other organisms, besides nuclear targets, cell wall integrity signaling pathways have cytoplasmic targets such as calcium channels, MAP kinase phosphatases and tyrosine phosphatases^13^. However, no cytoplasmic targets have been characterized in the cell wall integrity signaling pathway in *M*. *oryzae*.

GSK3 is well-known for its varied functions in regulation of cellular events in eukaryotic organisms^14^. A recent study in *F*. *graminearum* showed the sole GSK3 homolog Fgk3 had pleiotropic effects in fungal development and pathogenesis^20^. Fgk3 is involved in stress response via physical interaction with the stress-responsive transcriptional activator Msn2. After sensing the environmental cues, signaling cascades in plant pathogens are triggered and promote the differentiation of infection-related structures on plant surface^33^. Appressorium formation and penetration are nesessary for infection of *M*. *oryzae*. Gene targeted replacement of *MoGSK1* caused the production of non-functional hypha-driven appressoria, resembling the effect of *MPS1* deletion on appressorium development (Fig. 4). As a constitutively active kinase, regulatory role of GSK3 is usually through its inactivation after being subject to phosphorylation by upstream kinases^16^. This suggests that expression of *MoGSK1* may be targeted and suppressed by the Mps1 signaling pathway following the recognition of environmental cues, causing the subsequent unleash of GSK3 downstream targets and activation of genes related to cell wall biogenesis in matured appressoria. On the contrary, overexpression of *MoGSK1* produced elongated appressoria, which also supports the timely regulated *MoGSK1* signaling output is critical for proper appressorium development (Fig. 6).

As a central regulator in various cellular events, MoGsk1 has extensive roles in growth and development of *M*. *oryzae*. Deletion of *MoGSK1* significantly reduces the mycelial growth and abolishes fungal conidiation (Fig. 3), which are more severe compared to phenotypes observed on the *Δmps1* mutant^10^. This suggests that MoGsk1 is also involved in other signaling pathways more than the Mps1-mediated pathway. Pleiotropic effects of GSK3 in fungal life cycle has been observed in *F*. *graminearum* ^20^. Expression of *Fgk3* in *Δmogsk1* has comparable effect with *MoGSK1* in the complementation assays and shows the similar cytoplasmic localization in conidia (Fig. 5). Together with the cold-sensitivity rescue effect of *MoGSK1* in *S*. *cerevisiae Δmck1* (Fig. 2), these results suggest GSK3 members in fungi are structurally conserved and share fundamental functionalities across related species. Thus, the role of MoGsk1 is more about the regulation of the cell wall integrity pathway.

In budding yeast *S*. *cerevisiae*, Mck1 was also identified as a component of the Ca^2+^ signaling pathway that down-regulates Hsl1, a morphogenetic checkpoint protein kinase controlling proper timing of septin collar formation at mother-bud neck^34, 35^. In *M*. *oryzae*, a septin-forming ring at the appressorium base has been observed to scaffold a toroidal F-actin network that is necessary for penetration of leaf cuticles^36^. However, both septin ring and F-actin network were absent in *Δmps1*, contributing to the penetration defect in *Δmps1* ^36^. This may indicate that MoGsk1 mediates another signaling pathway to facilitate timing and establishment of appressorium morphogenesis. Elongated appressoria detected in the *MoGSK1* over-expression strain also suggests a link between the morphogenesis checkpoint and MoGsk1 (Fig. 6).

Another possible role of MoGsk1 is upon on its regulation of glycogen metabolism that is important for pathogenic progression in *M*. *oryzae* ^37^. In *F*. *graminearum*, conidia of *Δfgk3* accumulates more glycogen than the wild type strain^20^, suggesting an unbalanced glycogen homeostasis. Given the close relatedness between two GSK3 members of *M*. *oryzae* and *F*. *graminearum* (Fig. 5), it is plausible that MoGsk1 is involved in glycogen metabolism. It is noted that, rather than serving as a nutrient source, glucose and its derivates play roles as signal molecules in mediation of the invasive growth of *M*. *oryzae* via the modulation of the NADPH-dependent genetic switch^37, 38^. The protein kinase MoYAK1 responsible for modulating signal pathways triggered in response to surface hydrophobicity, glycogen status and cell wall integrity exert profound impacts on fungal growth, conidiation and infection-related development in *M*. *oryzae* ^39^. This confirms that protein kinases functioning in transmitting extracellular stimuli are able to regulate a diverse array of downstream pathways. Therefore, the MoGsk1-centring regulatory network involved in infection-related development in *M*. *oryzae* remains to be clarified. In the future, it will be important to determine the physical downstream targets of MoGsk1 and reveal their roles in different aspects of fungal development and pathogenesis.

## Methods

### Fungal strains and growth condition

Media composition, fungal growth, nucleic acid extraction and DNA-mediated transformation were all carried out as described previously^40^. Restriction digests, gel electrophoresis, and DNA and RNA gel blot hybridizations were all carried out using standard procedures^41^.

### Complementation of *S*. *cerevisiae Δmck1* with *MoGSK1*

The 1.2 kb *MoGSK1* cDNA was amplified using the Thermo Start Master Mix (Thermo Fisher Scientific), and ligated into the pYES2 vector (Invitrogen) using primers (GSK1YESF and GSK1YESR) resulting in the construct *pYES-MoGSK1*. Positive clones were confirmed by restriction digest and transformed into the *S*. *cerevisiae Δmck1* strain (BY4741; Mata; his3Δ1; leu2Δ0; met15Δ0; ura3Δ0; YNL307c::kanMX4) (EUROSCARF). The transformation of *S*. *cerevisiae* was carried out as described by^42^ using the standard high efficiency transformation protocol.

### *MoGSK1* gene targeted replacement and complementation

In order to knock out *MoGSK1*, a 1142 bp upstream fragment of the *MoGSK1* ORF in *M*. *oryzae* genome was amplified using primers GSK11F and GSK11R, and cloned into the *Kpn*I and *Hind*III sites on pCX62 generating the resulting construct pCX63. Then a 1267 bp fragment at the downstream of *MoGSK1* ORF was amplified using primers GSK12F and GSK12R, and then cloned into *Spe*I and *Sac*I sites on pCX63 resulting in the *MoGSK1* ORF replacement construct pCX64, which contained the *hygromycin phosphotransferase gene* (*hph*) as the selective marker flanked by two *MoGSK1* ORF flanking sequences. pCX64 was then transformed into Ku80 protoplast. Transformants with hygromycin resistance were screened by PCR with primers GSK13F and GSK13R to detect *MoGSK1* ORF, and GSK14F and GSK14R to detect the fragment spanning over *hph* gene and 5′ end flanking sequence of *MoGSK1* ORF. To rescue the *Δmogsk*1 phenotypes, a 7253 bp DNA fragment containing the native promoter, *MoGSK1* ORF and 3′ end UTR was amplified using primers GSK15F and GSK15R. The complementary strain was generated by co-transforming the PCR fragment with the *neomycin-resistant gene* (*neo*) containing vector pKNTG into *Δmogsk1* protoplast. Transformants were screened on neomycin containing plates and confirmed by Southern blot analysis.

### Characterization of the *Δmogsk1* mutant

Mycelial plugs of Ku80 and *Δmogsk1* were inoculated on CM medium in 9 cm diameter petri dish, the diameter of fungal colonies were measured after 3, 5, 7 and 10 days inoculation with three replicates. The capability to produce conidia was measured by calculating conidia collected in 10-day-old cultures on oatmeal agar media with consistent exposure to light per pertri dish plate. The observation on conidial development from conidiophores was performed as follows. The mycelia on 5-day-old oat meal cultures were pushed down with a glass slide. A 5 mm thick oat meal agar block was out from the edge of the colony and placed on a glass slide and incubated in a moisture chamber exposed to continuous light for 48 hours. Development of conidia was then examined by a light microscope (Zeiss Axioplan). In order to stain the conidiophores, an aqueous of oat meal agar was placed on a sterile glass slide, following the inoculation of fungal strains on oat meal agar. The glass slides were kept in a moist chamber under continuous light for 4 days. The agar block was then removed, and stained using a drop of 0.1% lactophenol aniline blue for 3 minutes before a cover slip was applied on. The slide was observed under a light microscope (Zeiss Axioplan).

### Infection assays with rice and barley leaves

Equal amounts of mycelial plugs (1 cm in diameter) were sliced out from the edge of 3-day-old colony on CM medium culture, and then inoculated on 7-day-old susceptible barley seedlings (CDC Silky) and 15-day-old rice seedlings (CO39). Lesion formation was examined after 5 days’ inoculation. To observe the appressorium penetration and invasive hyphae growth, 48 hours old inoculated barley leaves were sampled and fixed in a fixation solution (60% methanol, 30% chloroform, 10% acetic acid) for decolorization. Fixed samples were then rehydrated with reduced gradient ethanol (95%, 75%, 50% and 25%) and distilled water. Appressoria and invasive hyphae were then examined by a light microscope (Zeiss Axioplan).

### Subcellular localization and complementation of MoGsk1 and Fgk3

To construct *MoGSK1-GFP*, a DNA fragment containing the 5 kb promoter region, and 1.6 kb *MoGSK1* ORF were PCR-amplified using primers (MoGSK1/NF and MoGSK1-GFP/R) from Ku80. To construct *Fgk3-GFP*, the 5 kb *MoGSK1* promoter region was amplified using primers (MoGSK1/NF and MoGSK1/NR) from Ku80. The 1.2 kb *Fgk3* ORF region was amplified using primers (Fgk3/OF and Fgk3-GFP/R) from wild type *F*. *graminearum* PH-1 cDNA and ligated at 3′ end of the *MoGSK1* promoter region via overlap PCR approach. Both PCR-amplified fragments of *MoGSK1* and *Fgk3* under the same *MoGSK1* promoter were cloned into *Kpn*I and *Hind*III double-digested GFP-contained pKNTG by an *in vitro* recombination approach using the pEASY-Uni Seamless Cloning and Assembly Kit (TransGen Biotech, China). The resulting construct *pKNTG-MoGSK1-GFP* and *pKNTG-Fgk3-GFP* were transferred into *Δmogsk1* protoplasts, respectively.

### Creation of *MoGSK1* over-expression strain *P*_*MPG1*_*:MoGSK1*

The *MoGSK1* over-expression vector was created by fusing a 1.3 kb fragment of the *MPG1* promoter^43^ with the *MoGSK1* gene. A 1.9 kb genomic *MoGSK1* fragment was amplified using a forward primer GSK1OEF designed to span the ATG translation initiation codon and a reverse primer GSK1OER after the stop codon. The 1.3 kb *MPG1* promoter had been previously amplified and cloned into pGEM-5Z^®^, to form pNJT190^44^. The 1.9 kb genomic fragment spanning the *MoGSK1* locus was amplified by PCR using Thermo Start Master Mix (Thermo Fisher Scientific) and ligated into pNJT190. The complete 3.2 kb *P*_*MPG1*_*:MoGSK1* construct was removed from pNJT190 and ligated into pCB1004. The resulting construct *pCB1004-P*_*MPG1*_*:MoGSK1* was transformed into Guy11 protoplasts and screened on hygromycin B-containing plates. The expression of *MoGSK1* was tested by Northern blot analysis using a 1 kb *MoGSK1* fragment as the probes amplified from Poly (A)+ purified cDNA.

All primers are listed in Table S1.

## Electronic supplementary material

Supplementary information
