# Publisher Correction: The glycogen synthase kinase MoGsk1, regulated by Mps1 MAP kinase, is required for fungal development and pathogenicity in *Magnaporthe oryzae*

**DOI:** 10.1038/s41598-020-58148-7

**Published:** 2020-01-21

**Authors:** Tengsheng Zhou, Yasin F. Dagdas, Xiaohan Zhu, Shiqin Zheng, Liqiong Chen, Zachary Cartwright, Nicholas J. Talbot, Zonghua Wang

**Affiliations:** 1 Fujian-Taiwan Joint Center for Ecological Control of Crop Pests, Fujian Agriculture and Forestry University, Fuzhou, 350002 China; 2 Fujian University Key Laboratory for Functional Genomics of Plant Fungal Pathogens, Fujian Agriculture and Forestry University, Fuzhou, 350002 China; 3 School of Biosciences, University of Exeter, Exeter, EX4 4QD UK

Correction to: *Scientific Reports* 10.1038/s41598-017-01006-w, published online 19 April 2017

This Article contains errors in Figures 4A, 4B and 6B, where the scale bars are missing. The correct Figures 4 and 6 appear below as Figures 1 and 2 respectively.

**Figure 1:**
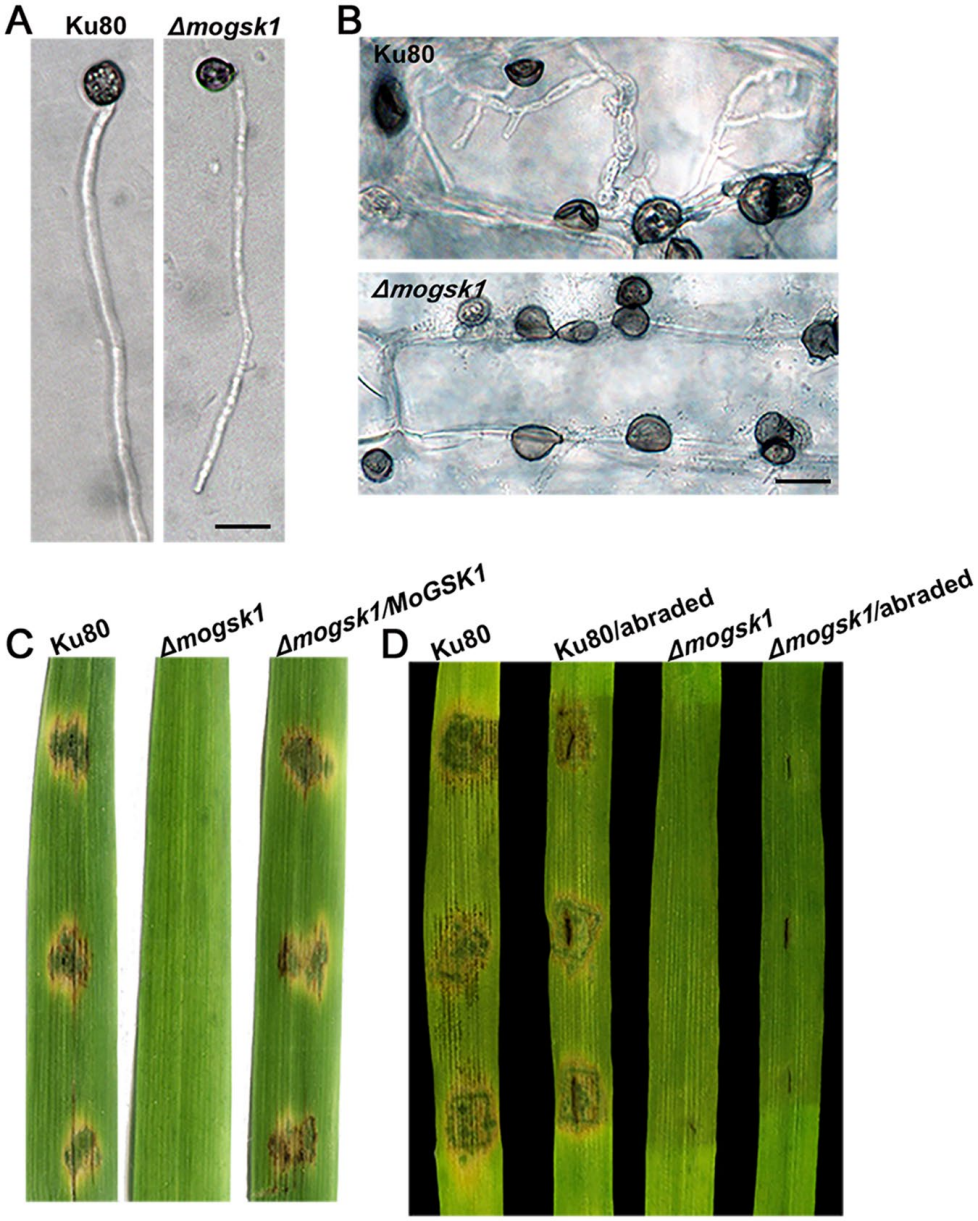
Plant infection assays and microscopic observation on infection process of the *Δmogsk1* mutant. (**A**) Appressoria of Ku80 and *Δmogsk1* were induced at the hyphal tips following 48 h inoculation on hydrophobic cover slips at a moisture chamber at room temperature. Bar = 10 μm. (**B**) Microscopic observation on mycelial plug inoculated area on unwounded barley leaf tissues 48 hr post inoculation. Bar = 10 μm. (**C**) Equal amount of mycelial plugs from Ku80, *Δmogsk1* and *Δmogsk1/MoGSK1* were inoculated on 15-day-old rice seedlings (CO39). Photos were taken post 5-day inoculation. (**D**) Disease symptoms on wounded and unwounded 7-day-old susceptible barley seedlings induced by mycelia plugs of Ku80 and *Δmogsk1* were photographed post 5-day inoculation.

**Figure 2:**
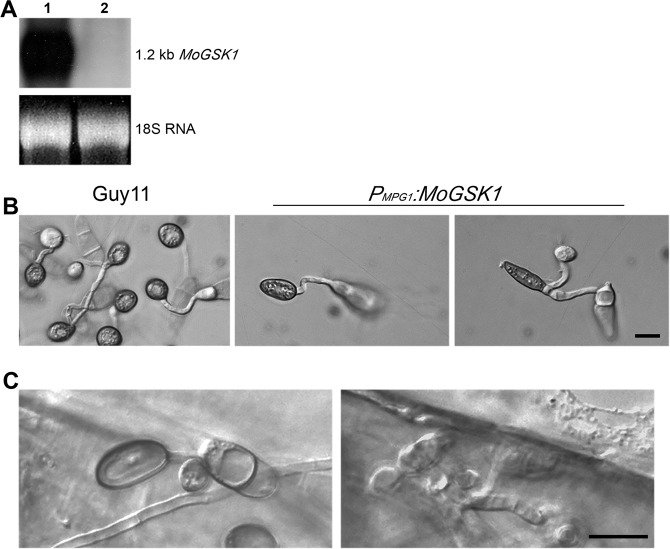
Over-expression of *MoGSK1* affects appressorium morphogenesis in *M*. *oryzae*. (**A**) RNA gel blot showing induction of *MoGSK1* (Line 1) in the transformant expressing *P*_*MPG1*_:*MoGSK1* compared to Guy11. (**B**) Microscopic observation of appressorium morphology induced on hydrophobic cover slips for 24 hr in the transformant expressing *P*_*MPG1*_*:MoGSK1*. Bar = 10 μm. (**C**) Penetration assay to demonstrate pathogenicity of the *MoGSK*1 overexpression strain. Appressorium formation (24 hr) and penetration hyphae (48 hr) developed on plant surface are shown in left and right hand panels. Bar = 10 μm.

