# Supplementary material for: The glycogen synthase kinase MoGsk1, regulated by Mps1 MAP kinase, is required for fungal development and pathogenicity in Magnaporthe oryzae
Source: Sci Rep. 2017 Apr 19;7:945. doi: 10.1038/s41598-017-01006-w (PMC5430414; doi:10.1038/s41598-017-01006-w)
Supplement: Supplementary file 1 — Supplementary information [file 41598_2017_1006_MOESM1_ESM.pdf]

**The glycogen synthase kinase MoGsk1, regulated by Mps1 MAP kinase, is required for fungal development and pathogenicity in *Magnaporthe oryzae***

**Tengsheng Zhou[1,2*], Yasin F. Dagdas[3*], Xiaohan Zhu[1,2], Shiqin Zheng[1], Liqiong Chen[2], Zachary Cartwright[3], Nicholas J. Talbot[3#] and Zonghua Wang[1,2#]**

**[1] Fujian-Taiwan Joint Center for Ecological Control of Crop Pests, Fujian Agriculture and Forestry University, Fuzhou, 350002, China.**

**[2] Fujian University Key Laboratory for Functional Genomics of Plant Fungal Pathogens, Fujian Agriculture and Forestry University, Fuzhou, 350002, China.**

**[3] School of Biosciences, University of Exeter, Exeter EX4 4QD, United Kingdom**

*** These authors contributed to the work equally**

**# Corresponding authors: n.j.talbot@exeter.ac.uk, wangzh@fafu.edu.cn**

```
MoGSK1     1 MSQN--RPAAFNTLR-MGEVIREKVQDGITGETRDLQYTQCKIVGNGSFGVVFQTKLSPS
CgGSK1     1 MAQN--RPAAFNTLR-MGEVIREKVQDGITGETRDLQYTQCKIVGNGSFGVVFQTKLSPS
Fgk3       1 MSAH--RPNAFNSLR-MGEVIREKVQDGITGETRDLQYTQCKIVGNGSFGVVFQTKLSPS
UmGSK1     1 MSNAPLNGVKLNPLDDPNKVIKVLASDGKTGEQREIAYTNCKVIGNGSFGVVFQAKLVSQ
ScMCK1     1 MSTEEQNGVPLQRGS---EFIADDVTSNKSNNTRRMLVKEYRKIGRGAFGTVVQAYLTQD

MoGSK1    58 N-----------EDAAIKRVLQDKRFKNRELQIMRIVRHPNIVQLKAFYYSNGERKDEVY
CgGSK1    58 G-----------EDAAIKRVLQDKRFKNRELQIMRIVRHPNIVQLKAFYYSNGERKDEVY
Fgk3      58 G-----------EDAAIKRVLQDKRFKNRELQIMRIVRHPNIVQLKAFYYSNGERKDEVY
UmGSK1    61 GSEPAEGSSKESDEVAIKKVLQDKRFKNRELQIMRIVKHPNVVDLKAFFYSNGDKKDEVF
ScMCK1    58 KK-------NWLGPFAIKKVPAHTEYKSRELQILRIADHPNIVKLQYFFTHLSPQDNKVY

MoGSK1   107 LNLVQEFVPETVYRASRFFNKMKTTMPILEVKLYIYQLFRALAYIHSQGICHRDIKPQNL
CgGSK1   107 LNLVQEFVPETVYRASRFFNKMKTTMPILEVKLYIYQLFRALAYIHSQGICHRDIKPQNL
Fgk3     107 LNLVQEFVPETVYRASRFFNKMKTTMPILEVKLYIYQLFRALAYIHSQGICHRDIKPQNL
UmGSK1   121 LNLVLEYVPETVYRASRHYAKLKQTMPMLLIKLYMYQLLRSLAYIHSIGICHRDIKPQNL
ScMCK1   111 QHLAMECLPETLQIEINRYVTNKLEMPLKHIRLYTYQIARGMLYLHGLGVCHRDIKPSNV

MoGSK1   167 LLDPTTGILKLCDFGSAKILVENEPNVSYICSRYYRAPELIFGATNYTTKIDVWSTGCVM
CgGSK1   167 LLDPTSGILKLCDFGSAKILVENEPNVSYICSRYYRAPELIFGATNYTTKIDVWSTGCVM
Fgk3     167 LLDPNSGILKLCDFGSAKILVPNEPNVSYICSRYYRAPELIFGATNYTTKIDVWSTGCVM
UmGSK1   181 LLDPPSGVLKLIDFGSAKILIAGEPNVSYICSRYYRAPELIFGATNYTTNIDIWSTGCVM
ScMCK1   171 LVDPETGVLKICDFGSAKKLEHNQPSISYICSRFYRAPELIIGCTQYTTQIDIWGLGCVM

MoGSK1   227 AELMLGQPLFPGESGIDQLVEIIKVLGTPTREQIRTMNPNY-----MEHKFPQIKPHPFN
CgGSK1   227 AELMLGQPLFPGESGIDQLVEIIKVLGTPTREQIRTMNPNY-----MEHKFPQIKPHPFA
Fgk3     227 AELMLGQPLFPGESGIDQLVEIIKVLGTPTREQIRTMNPNY-----MEHKFPQIKPHPFN
UmGSK1   241 AELMQGQPLFPGESGIDQLVEIIKVLGTPSREQIKTMNPNY-----MEHKFPQIRPHPFS
ScMCK1   231 GEMLIGKAIFQGQEPLLQLREIAKLLGPPDKRFIFFSNPAYDGPLFSKPLFSGSSQQRFE

MoGSK1   282 RVLR-KADNNAIDLIARLLEYTPTERLGAIDAMVHPFFDDLRNPSTKLPDSRHQTGQVRD
CgGSK1   282 KVFR-KADANAIDLIARLLEYTPTERQAAVEAMIHPFFDELRDPNTKLPDSRHQSGEVRD
Fgk3     282 KVFR-KADANAIDLIARLLEYTPTERQSAIDAMVHPFFDELRDPNTKLPDSRHGTGQLRD
UmGSK1   296 KVFRPRTPPDAIDLISRLLEYTPSARLTAIEALCHPFFDELRTGEARMPNG-------RE
ScMCK1   291 KYFG-HSGPDGIDLLMKILVYEPQQRLSPRRILAHQFFNELRNDDTFLPRG---FTEPIK

MoGSK1   341 LPPLFDFNRHELSIAPQLNHQLVPPHVRPTLAAQGLDIDHFTPMRKEDMLARLD
CgGSK1   341 LPPLFDFTRHELSIAPNLNHQLVPPHMKPVLAGRGLDIDNFTPIPKADMMAKLD
Fgk3     341 LPALFDFTRHELSIAPSLNPKLVPAHIRPVLASQGLDIDHFTPLTEQEMMAKLD
UmGSK1   349 LPPLFNWTKEELSVRPDLISRLVPQHAEAELLSRGIDVHNFQPIPLESLKVTLD
ScMCK1   347 LPNLFDFNDFELQILGEFADKIKPTKVAE-------------------------
```

**Figure S1**. Alignment of the *M. oryzae* MoGSK1 with identified or hypothetical members of glycogen synthase kinase 3 from other fungi. The sequenced *M. oryzae* MoGSK1 was aligned with: putative CgGSK1 from *Colletotrichum graminicola* (GenBank accession XP_008091917), identified Fgk3 (XP_009255527) from *Fusarium graminearum*, putative UmGSK1 from *Ustilago maydis* (XP_011386391), and identified ScMCK1 (NP_014092) from *Saccharomyces cerevisiae*. The ClustalW program was used to align the sequences. Identical amino acid residues are indicated in background, similar residues are indicated in gray background.

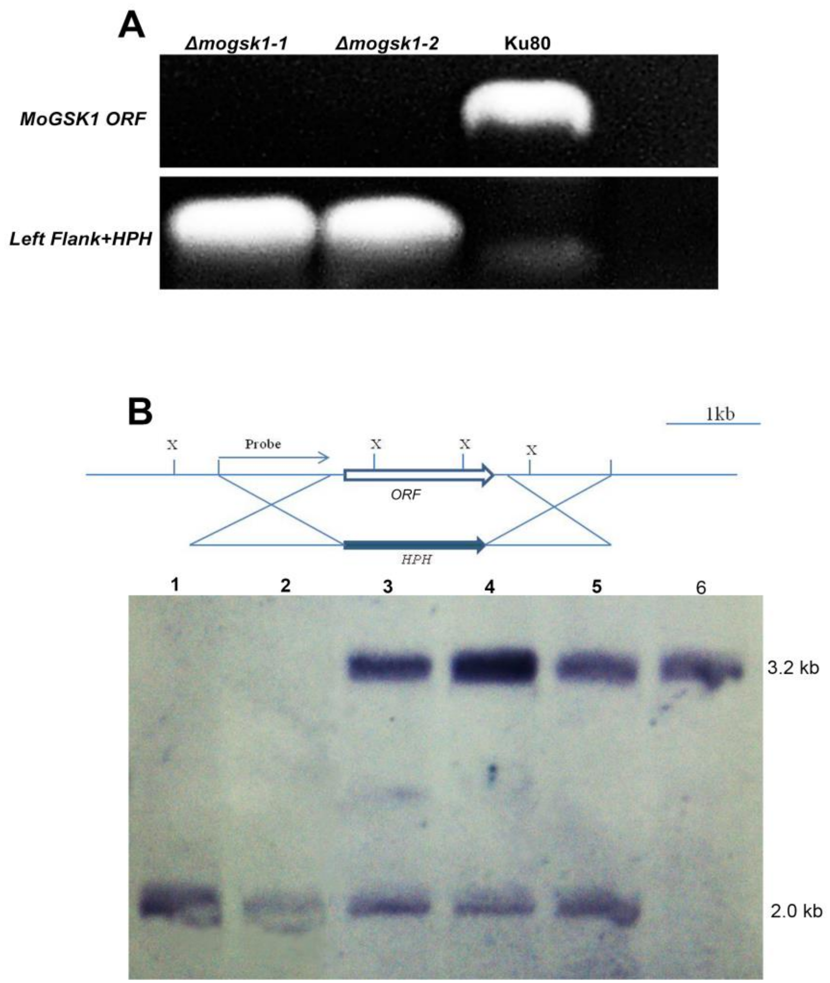


Figure S2. Confirmation of the *Δmogsk1* mutant. (A) Total genomic DNA isolated from mycelia of Ku80 and two gene deletion line *Δmogsk1-1* and *Δmogsk1-2* were subjected to PCR analysis by using *MoGSK1* specific primers (GSK13F and GSK13R) and *hph* primers (GSK14F and GSK14R). (B) DNA gel blot analysis. Genomic DNA was collected from mycelia of (Lane 1) *Δmogsk1-1*, (Lane 2) *Δmogsk1-2*, and the complementation transformants (Lane 3) *MoGSK1-com*-118, (Lane 4) *MoGSK1-com*-126， (Lane 5) *MoGSK1-com*-132， and (Lane 6) Ku80 strains and digested with *XhoI*I and blotted using the probe amplified by GSK15F and GSK15R. The results show a single 2042 bp band from *Δmogsk1-1*, *Δmogsk1-2*, *MoGSK1-com*-118, *MoGSK1-com*-126, *MoGSK1-com*-132 and a single 3265bp band from *MoGSK1-com*-118, *MoGSK1-com*-126, *MoGSK1-com*-132 and Ku80.

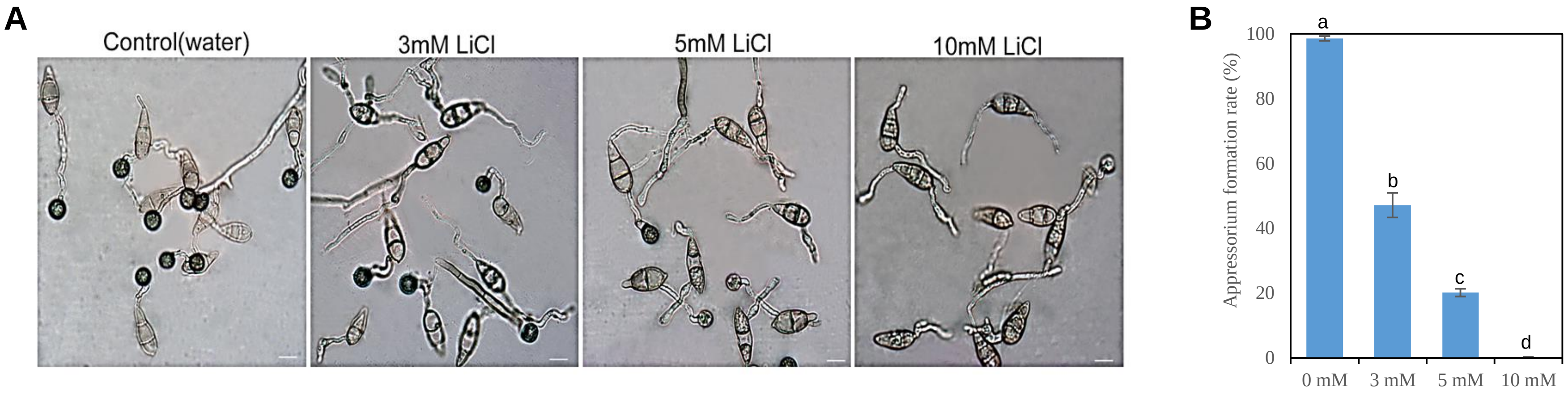


Figure S3. The treatment of LiCl on appressorium development in *M. oryze*. (A) Aliquots of 10 uL Conidia suspension (1×10$^4$ conidia/mL) of wild type Guy11 supplemented with $H_2O$ and LiCl at concentration of 3mM, 5mM and 10mM were inoculated on hydrophobic cover slips to form appressoria. Photos were taken 16 hours post inoculation (Bar = 10 μm). (B) Average percentage of appressorium formation rate after Licl treatment on hydrophobic cover slips. Data are shown as means ±SD, n = 3, α = 0.01 (ANOVA).

**Table S1. Detailed information of primers used in this study.**

| Primer name | Sequence (5' -3') | Application |
|---|---|---|
| GSK1YESF | GCGAATTCGCTGCCCGCGAT GTCTCAGA | Yeast complementation |
| GSK1YESR | GCACTAGTAACACCACCGAC AGTCGAGG | Yeast complementation |
| GSK11F | CGGGGTACCGCGGTTTCCAT TTGTGA | Targeted gene deletion |
| GSK11R | CCCAAGCTTGTGTCCGTCGTC TCGTT | Targeted gene deletion |
| GSK12F | GGACTAGTGGGCTCCCATAG CTCATA | Targeted gene deletion |
| GSK12R | CGGAGCTCCACTCACTCCTG CCAACT | Targeted gene deletion |
| GSK13F | ACGAAGATGCTGCGATAA | Targeted gene deletion |
| GSK13R | TCTGAGGGAACTTGTGCT | Targeted gene deletion |
| GSK14F | GTCTGCACCCGTCATCC | Targeted gene deletion |
| GSK14R | TGTTGGCGACCTCGTATT | Targeted gene deletion |
| GSK15F | CCCAAGCTTTACCATTTGGCG GTTTGC | Mutant complementation |
| GSK15R | CGGAATTCAGCATCACTCCA GCGGGTC | Mutant complementation |
| GSK1OEF | GCTCATGACTCAGAACCGTC CAGCAG | Gene overexpression |
| GSK1OER | GCGGGCCCAACACCACCGAC AGTCGA | Gene overexpression |
| MoGSK1/NF | AGGGAACAAAAGCTGGGTAC CTACCATTTGGCGGTTTGC | MoGSK1 subcellular localization |
| MoGSK1-GFP/R | GAACAGCTCCTCGCCCTTGCT CACATCTAACCTTGCAAGCA TGTCCTC | MoGSK1 subcellular localization |

| | | |
|---|---|---|
| MoGSK1/NR | TTGGCCGATGAGCCGACATCGCGGGCAGCCGACTCGATTTTCTCCAA | Fgk3 subcellular localization |
| FgGSK3/OF | TCGAGTCGGCTGCCCGCGATGTCGGCTCATCGGCCAAAC | Fgk3 subcellular localization |
| FgGSK3-GFP/R | GAACAGCTCCTCGCCCTTGCTCACATCCAGTTTCGCCATCATCTCCT | Fgk3 subcellular localization |
